# Immune checkpoints in rheumatoid arthritis: progress and promise

**DOI:** 10.3389/fimmu.2023.1285554

**Published:** 2023-11-24

**Authors:** Annabelle Small, Katie Lowe, Mihir D. Wechalekar

**Affiliations:** ^1^ Department of Rheumatology, College of Medicine and Public Health, Flinders University, Adelaide, SA, Australia; ^2^ Department of Rheumatology, Flinders Medical Centre, Adelaide, SA, Australia

**Keywords:** rheumatoid arthritis, T cells, immune checkpoints, co-stimulatory pathways, CTLA-4, PD-1

## Abstract

Rheumatoid arthritis (RA) is one of the most prevalent autoimmune inflammatory conditions, and while the mechanisms driving pathogenesis are yet to be completely elucidated, self-reactive T cells and immune checkpoint pathways have a clear role. In this review, we provide an overview of the importance of checkpoint pathways in the T cell response and describe the involvement of these in RA development and progression. We discuss the relationship between immune checkpoint therapy in cancer and autoimmune adverse events, draw parallels with the involvement of immune checkpoints in RA pathobiology, summarise emerging research into some of the lesser-known pathways, and the potential of targeting checkpoint-related pathways in future treatment approaches to RA management.

## 1 Introduction

Rheumatoid arthritis (RA) is a chronic, systemic, inflammatory autoimmune disease that affects up to 1% of the population (1). Inflammation predominantly targets the synovial tissue (ST; the joint lining), leading to pain, swelling and irreversible joint deformities. Disease complications are not confined to joints, and include systemic involvement (pulmonary, cardiac, haematological, amongst others). Despite treatment advances, a majority of patients do not attain remission, and sustained remission occurs in as few as 15% of patients (2). Although the eventual inflammatory milieu in the RA ST is diverse, checkpoint, or co-stimulatory pathways controlling immune cell activation are critical in engendering, maintaining, and perpetuating the inflammatory response, driven by chronic autoimmune antigenic drive. In parallel with the rise of immune checkpoint inhibitors (ICIs) that have transformed oncologic therapeutics, has been the greater recognition of checkpoint pathways in RA pathogenesis.

In this review, we provide an overview of immune checkpoints at various stages of immune cell development and describe the involvement of these in the development and progression of RA. We discuss the involvement of immune checkpoints in RA pathobiology, the relationship between immune checkpoint therapy in cancer and autoimmune adverse events, and the potential to manipulate these pathways as a future treatment strategy.

## 2 Immune checkpoints: dictators of the T cell response

**Figure 1:**
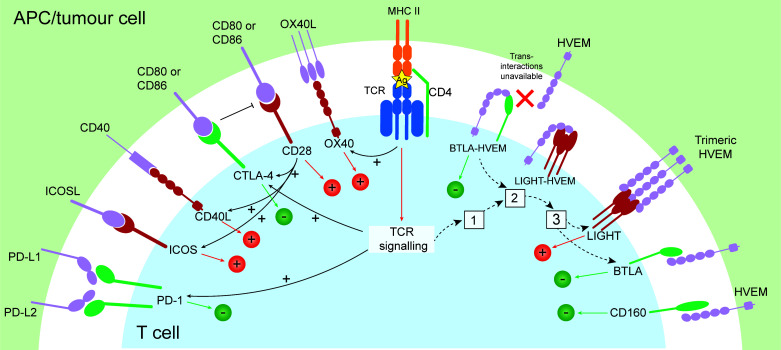
Co-signalling pathways with either stimulatory (+) or inhibitory function (-), and the relationship between their expression. Following presentation of antigen by APC or tumour cell and recognition by a T cell expressing a cognate TCR and co-stimulation provided through CD28 binding CD80/CD86, stimulatory (OX40, CD40L, ICOS, LIGHT) and inhibitory checkpoints are induced (CTLA-4, PD-1). In the resting state, BTLA and HVEM form a *cis-* complex which exerts constitutive inhibitory signalling to the T cell. Following T cell activation, LIGHT is rapidly and transiently induced (1), and LIGHT disrupts BTLA-HVEM complexes, sequestering HVEM from BTLA. This results in internalisation of HVEM (2), and leaves surface LIGHT and BTLA available for *trans-* interactions with HVEM (3), capable of inducing stimulatory or inhibitory signals, respectively. Ag, antigen.

T cell activation is a tightly regulated, intricate, and specific process requiring multiple initiating signals. The first is provided through the interaction between a peptide antigen presented by an antigen presenting cell (APC), and a T cell expressing a cognate T cell receptor (TCR). The T cell then requires a secondary signal, referred to as co-stimulation, and the proteins that provide this are called co-stimulatory, or checkpoint molecules. In helper T cells, this is provided through the B7/CD28 co-stimulation axis (3). CD28 expressed on the T cell binds to either CD80 (B7.1) or CD86 (B7.2) on the APC, and together with TCR signalling, provides survival and activation signalling to initiate T cell proliferation (**Figure 1**). The extent of co-stimulation controls the extent of the initial T cell proliferative response (4), and further signalling provided through specific cytokines dictates cell lineage (5).

Checkpoint molecule expression is largely controlled by the tissue microenvironment, and is influenced by alterations in homeostasis (6). These molecules can exert stimulatory or inhibitory signals in either direction of the interaction (i.e., in the APC and in the T cell) and cumulatively dictate the threshold of T cell activation, allowing for finetuning of the immune response (7, 8). Inhibitory checkpoints, including cytotoxic T-lymphocyte-associated protein 4 (CTLA-4) and programmed death protein 1 (PD-1) are upregulated during cell activation, and are important for limiting the response duration, aiding resolution, and maintaining self-tolerance (7). The importance of immune checkpoints in restricting the immune response is exemplified by disturbances in these pathways. Patients with inherited CTLA-4 deficiencies present with a range of clinical features including recurrent respiratory tract infections and increased risk of autoimmunity (9), while inherited PD-1 deficiency has been linked with early-onset autoimmunity and severe tuberculosis (10). Mice deficient in CTLA-4 experience lethal lymphoproliferative disorders (11), and those deficient in PD-1 develop accelerated autoimmunity and lethal pathology in response to acute infection (12–14).

## 3 The T cell response in RA

The importance of the T cell response in RA has long been appreciated (15–18) and evidence suggests T cell-driven autoimmunity may occur years prior to clinical onset (19, 20). Distinct changes have been observed in the proportions of T follicular helper (Tfh) and CD19^+^ B cells in the lymph nodes of patients at risk of developing RA (individuals with rheumatoid factor (RF) and anti-citrullinated protein antibodies (ACPA) but asymptomatic for arthritis) (21), and altered proportions of naïve T cells and T regulatory cells (Treg) have been observed in the peripheral blood (22). In lymph nodes, stromal cells from at-risk patients had distinct hypomethylated sites in genes associated with antigen processing and presentation compared to healthy controls, suggesting altered antigen presentation pathways prior to clinically identifiable disease (23), implicating stromal-lymphoid cell interactions in early RA. Finally, it is well established that RF and ACPA can often be detected in the serum for years in individuals who progress to develop inflammatory arthritis (20, 24).

In clinically identifiable early and established RA, T cells accumulate in the ST sublining layer, where they’re found in association with activated macrophages and B cells (16), and often cluster in aggregates consisting of T cells, B cells, and dendritic cells (DC) (17). While there is enormous variability in synovitis in RA (17, 25), evidence suggests a T helper 1 (Th1) bias in the ST (17), and the demonstration of T cell involvement across all stages of disease suggests that T cell targeting may be efficacious therapeutically broadly throughout disease progression.

Aligning with the importance of the T cell response in RA progression, CTLA-4 fusion protein (CTLA-4-Ig; Abatacept), a soluble protein that binds CD80/CD86 expressed by activated APCs and outcompetes CD28 expressed by T cells at the beginning of the immune response, was approved for use in RA in 2005. Abatacept therefore inhibits circulating and synovial T cell activation by inhibiting CTLA-4:CD28 interactions, and has shown remarkable efficacy in disease management (26), reducing progression of undifferentiated arthritis to RA, and reduction of radiological progression and levels of anti-CCP antibodies (27). Early outcomes from two ongoing trials further suggest Abatacept may also prevent RA development in at-risk healthy individuals (28–30).

## 4 Inflammatory arthritis as an adverse event of checkpoint therapy in cancer

In cancer, tumour cells can exploit checkpoint pathways controlling the adaptive immune response to neoantigens, inducing tolerance and immune evasion (31, 32). ICIs targeting co-inhibitory receptors in the tumour microenvironment have had remarkable success by unleashing the anti-tumour function of T cells. However, ICI usage is complicated by the occurrence of immune-related adverse events (irAEs) which can present with broad manifestations varying by ICI type (33). Additionally, while combinations of ICIs have shown improved oncologic therapeutic response compared to monotherapies (34), the proportion of patients who developed treatment-related severe irAEs were markedly higher with combination therapy (55%) compared with those in the nivolumab- (16.3%) or ipilimumab-only groups (27.3%), demonstrating that combination therapy may dramatically increase irAE risk (34).

Rheumatological complications occur in ~5-10% of patients treated with ICIs (35), and can manifest in the form of joint inflammation indistinguishable from RA (36), which can persist after therapy cessation (37) and exacerbate existing RA symptoms (38). Additionally, inflammatory arthritis is the most common irAE reported in trials of ICIs (39), an occurrence that has offered a serendipitous window into RA pathogenesis.

## 5 The role of co-stimulatory pathways in RA

### 5.1 CTLA-4/CD28/CD80/CD86 pathway

The most well-studied checkpoint pathway in RA is the CTLA-4/CD28/CD80/CD86 pathway. CTLA-4 is the first co-inhibitory molecule to be upregulated following lymphocyte activation (**Figure 1**) and is critical for controlling initial naïve T cell activation in lymph nodes (40, 41). CTLA-4 is important for Treg function, and binds CD80 with approximately 20 times the affinity of CD28 (42), enabling it to potently inhibit T cell activation. Following binding to CD80/CD86, CTLA-4 can transendocytose its ligands from the APC which are then degraded by the T cell, further impairing co-stimulation through CD28 (43–45).

The most compelling evidence of the role of the CTLA-4/CD28 pathway in the pathogenesis of RA is demonstrated by the efficacy of Abatacept in treating RA. Accordingly, polymorphisms in *CTLA4* have been associated with both the development and a decreased risk of RA (46, 47). In RA, serum concentrations of soluble CTLA-4 and CD28 were higher than in healthy controls and serum CTLA-4 correlated positively with disease activity (DAS28) (48), while CTLA-4 expression on RA Tregs was lower compared with healthy controls (49). However, despite the clear role of the CTLA-4/CD28 pathway in the RA immune response, many patients do not respond adequately to Abatacept, indicating disease biology driven by alternative inflammatory pathways (possibly including other checkpoint mechanisms) and highlight the critical unmet need for the development of individualised targeted therapy in RA. Further research into the potential of targeting other co-stimulatory pathways may provide these new approaches, and our knowledge of the role of these pathways in RA is summarised below.

### 5.2 The PD-1 pathway

Inhibitory signalling through PD-1 regulates activated T cells in later stages of activation (**Figure 1**) primarily in peripheral tissues, and is associated with chronic antigen exposure (12, 40). PD-1 signalling results in immunoreceptor tyrosine-based switch motif phosphorylation and recruits the tyrosine phosphatase SHP-2, inhibiting cell proliferation and cytokine production (50). In the chronically inflamed RA synovium, in line with the relationship between PD-1/PD-L1 inhibitors and the development of irAEs, expression of PD-1 correlated with synovial inflammation (51), and PD-1 expressing T cells were enriched in the early and established RA ST and periphery (52, 53). These were T peripheral helper (Tph) cells, which contribute to pathogenesis by high production of CXCL13 and interleukin (IL)-21, enabling them to provide B cell help outside of germinal centres (53, 54). Tph cells express high levels of MAF and BATF, and unlike Tfh cells, low levels of *BCL6* (53). Additionally, accumulation of PD-1^+^ activated B cells characterised by increased CD80, CD86, IL-1β and GM-CSF have been demonstrated in the RA ST and SF (55).

Elevated soluble PD-1 (sPD-1) produced by alternative splicing has been demonstrated in the RA periphery (56), and sPD-1 levels positively correlated with RF titres (57) and levels of TNF in the synovial fluid (58). Meanwhile, the PD-1 ligand PD-L1 was conspicuously absent from the ST (56), and sPD-1 has been demonstrated to bind PD-L1 and PD-L2 (59). Therefore, the findings of elevated PD-1^+^ T cells in the synovium along with elevated sPD-1 and undetectable PD-L1 suggest that the PD-1 pathway is dysregulated in RA, and this lack of PD-1-mediated inhibitory signalling may result in chronic T cell activation, thus contributing to pathogenesis (58, 60).

The evidence suggesting PD-1 pathway dysregulation in RA and the identification of pathogenic PD-1-expressing cells, particularly in early RA, suggests the therapeutic potential of targeting this pathway. In a phase IIa trial, Peresolimab, an anti-PD-1 agonistic antibody, induced a significant reduction in DAS28-CRP by week 12 (61), while another PD-1 agonist Rosnilimab, is awaiting entry into phase II trials in the near future (62). However, given the success of PD-1 inhibition in cancer, longer phase III trials will be required to fully assess the safety profiles of these therapies with regards to oncologic risk.

Finally, recent studies have highlighted the interplay between the PD-1 and CTLA-4/CD28 pathways. On APCs, PD-L1 can form a complex in *cis* with CD80 (63). This duplex prevents PD-1:PD-L1 binding, inhibiting PD-1 function. Approaches to disrupt this complex using anti-CD80 antibody alleviated autoimmune symptoms (including experimental arthritis) in mice (64), and therefore further study into how the interplay between these pathways contributes to RA pathogenesis is warranted to determine whether similar approaches may be of therapeutic benefit.

### 5.3 CD40-CD40L pathway

CD40 ligand (CD40L) is a stimulatory checkpoint upregulated on T cells following activation. CD40-CD40L signalling promotes antibody production and class-switching in B cells (65), induces proinflammatory cytokines, chemokines, and adhesion molecules, and can regulate other immune checkpoints (66). Disruption of CD40-CD40L caused by inherited loss-of-function mutations in *CD40* or *CD40L* lead to the X-linked hyper-IgM syndrome, severely compromising humoral immunity (67). Conversely, gain-of-function mutations in *CD40* are associated with RA risk and patients homozygous for these alleles express increased B cell CD40 (68). Additionally, in undifferentiated arthritis, and early and established RA, expression of *CD40* and *CD40LG* were increased compared to osteoarthritis and healthy controls, while expanded CD8^+^CD40L^+^ ST T cells clones from RA patients expressed CD40L (69), further suggesting a role for increased local CD40- CD40L signalling in the RA ST (70). Finally, supporting the association between increased CD40-CD40L signalling and RA, a recent genome-wide protein quantitative trait locus (pQTL) study demonstrated association with elevated serum CD40 and RA risk (71).

While disruption of CD40-CD40L in patients with homozygous mutations is associated with immunodeficiency, heterozygous carriers are unaffected, indicating that therapeutic inhibition of CD40-CD40L may be well-tolerated (68). Indeed, in a phase 1b clinical trial assessing the therapeutic benefit of systemic CD40L blockade in RA, a CD40L-binding protein VIB4920 significantly decreased disease activity and demonstrated an acceptable safety profile (72). At week 12, 41.7% of patients in the VIB4920 1500 mg group achieved a DAS28-CRP score of <2.6, compared to 6.7% in the placebo group (72). Subsequently, VIB4920 has entered phase II trials alongside anti-TNF.

### 5.4 ICOS

The inducible T cell co-stimulator (ICOS) is expressed by T cells following CD28-co-stimulation (**Figure 1**) and is structurally similar to CD28, yet differs functionally by its ability to induce IL-10 following ligation, and inability to induce IL-2 (73). Interaction with its ligand ICOSL induces phosphoinositide 3-kinase (PIK3)-dependent signalling, promoting T cell differentiation and germinal centre formation (74). ICOS signalling was shown to be required for collagen induced arthritis (75, 76), and ICOS-ICOSL blockade targeting ICOSL significantly ameliorated joint inflammation, disease progression and severity (77). In RA, patients had increased proportions of ICOS^+^ synovial fluid T cells compared to RA peripheral blood T cells (78).

Acaziocolcept, a dual ICOS/CD28 inhibitor trialed for therapeutic efficacy in a murine model of systemic sclerosis (79), inhibited collagen-induced arthritis more potently than Abatacept, and inhibited human T cell function more effectively than inhibition of either ICOS or CD28 alone, suggesting its potential therapeutic use in human disease (80).

### 5.5 OX40L

OX40 (CD134), similarly to CTLA-4, PD-1 and ICOS, is predominantly expressed by T cells after activation (81). Its ligand OX40L can be induced on APC, Langerhans cells, endothelial cells, mast cells, and NK cells, suggesting a range of functions in lymphoid and non-lymphoid pathways (82). OX40 signalling on T cells provides synergistic signalling alongside CD80-CD28 that prolongs T cell proliferation and enhances IL-2 production (83), while bidirectional signalling through OX40L induces the production of proinflammatory mediators and increased antibody production (84).

In early RA, soluble OX40L was increased and positively correlated with ACPA and RF (85). In the joints, OX40L inhibited osteoclastogenesis (84), and mice lacking OX40L showed decreased bone integrity and enhanced osteoclastogenic capacity (84). Despite this, OX40L blockade in collagen induced arthritis reduced inflammation and inhibited inflammatory cytokine production by OX40L-expressing macrophages (84). Interestingly, patients with RA have been shown to have an abundance of OX40^+^ Tfh cells capable of producing IL-17 (86), and OX40 was enhanced on Tfh cells within the lymph nodes and demonstrated that severity of arthritis was reduced by blockade using anti-OX40L in glucose-6-phosphate isomerase-induced arthritis.

### 5.6 BTLA/HVEM/CD160/LIGHT regulatory network

B- and T-lymphocyte attenuator (BTLA) is a member of the CD28 immunoglobulin super family, and is expressed broadly by T and B cells, NK cells, and DCs (87). BTLA forms an intricate regulatory network together with CD160, and LIGHT (TNFSF14), sharing the ligand HVEM (herpes virus entry mediator; TNFRSF14) (88, 89). This network can elicit inhibitory or stimulatory signals depending on the cells expressing them and whether their interactions occur in *cis-* or *trans-* (**Figure 1**) (88). BTLA and HVEM were present in the established RA ST (90), while in the peripheral blood, BTLA was increased on circulating CD3^+^ T cells while HVEM and LIGHT were decreased compared to healthy controls (91). *BTLA* polymorphisms have also been associated with RA risk (92). HVEM also binds glycoprotein D (gD) which competes for binding with BTLA and CD160 (88, 93); efforts into blocking the BTLA-HVEM interaction using gD fragments for future cancer therapy are ongoing (94). Reciprocal approaches to free HVEM for interaction with BTLA and CD160 may induce inhibitory signalling and provide a future therapeutic approach in RA.

## 6 Other pathways

Research into other inhibitory immune checkpoints including TIGIT, TIM-3, and LAG-3 is currently underway in cancer (32, 95), and are tantalising future targets in RA. Increased TIGIT expression on CD4^+^ RA T cells correlated with autoantibody levels and DAS28 (96), and an agonistic anti-TIGIT antibody, capable of inhibiting Tfh and Tph cells and enhancing Treg function, has recently been described (97). Tregs expressing TIGIT supressed Th1 and Th17 cell responses (98) and signalling through TIGIT on functionally defective Tregs from patients with multiple sclerosis restored suppressive function (99), highlighting the potential of TIGIT-targeting. LAG-3 is constitutively expressed on Tregs; LAG-3 positive Tregs in peripheral blood were reduced in RA patients with higher disease activity, and increased following abatacept therapy (100). Additionally, soluble LAG-3 was increased in early and established RA, correlated with ACPA/RF status and erosive progression in early RA, and decreased inflammatory cytokine release in chronic RA (101). Finally, T cell expression of TIM-3, was increased in RA patients and correlated with decreased DAS28 (102).

Leukocyte-associated immunoglobulin receptors 1 and 2 (LAIR-1 and LAIR-2, respectively) are inhibitory immune receptors for collagens that are expressed broadly across the immune compartment (103, 104). LAIR-1 engagement by collagen or the complement component C1q induces inhibitory T cell signalling (105), and in collagen-induced arthritis, administration of anti-LAIR-1 antibodies significantly attenuated disease (106, 107). In RA, T cell expression of LAIR-1 is reduced compared to healthy and osteoarthritis controls and elevated on ST monocytes and macrophages (108). LAIR-2 is a secreted homolog capable of inhibiting LAIR-1-mediated inhibitory signalling, and is elevated in the RA synovial fluid (109), suggesting dysregulated LAIR-1 action in RA. Together, these suggest that manipulating inhibitory LAIR-1 signalling may offer a future therapeutic approach for RA.

VSIG4 (complement receptor immunoglobulin; CRIg) is a B7-family related protein capable of modulating T cells through interaction with a yet to be identified receptor and has additional roles in immunosuppression (110, 111). VSIG4 binds the complement fragment C3b, inhibiting the alternative complement pathway (112), and approaches utilising this function have shown efficacy in rodent models of RA (113, 114). Administration of soluble VSIG4 reduced disease severity and prevented bone erosion in murine arthritis models (114), and administration of the dual complement inhibitor CRIg-CD59 alleviated symptoms in an adjuvant-induced arthritis rat model (113). In humans, VSIG4 is expressed by tissue-resident regulatory macrophages in the healthy and RA ST from patients in remission (115), and importantly, polymorphisms in *VSIG4* have been associated with RA severity (116).

Finally, the signalling lymphocytic activation molecule (SLAM) family has gained recent attention in RA. SLAMF6 was increased in osteoarthritis and early RA ST (117), and in circulating early RA PD-1^+^CD4^+^ cells (54) and inhibition of SLAMF6 on CD4^+^PD-1^+^ peripheral cells from established RA patients decreased IgG production and plasmablast differentiation (53).

## 7 Future directions

With their undeniable role in the RA autoimmune milieu, the potential of targeting immune checkpoint pathways with novel approaches is an area of immense interest in RA research. These include modulation utilising small molecules including non-coding microRNAs, and their use in downregulating inhibitory checkpoint pathways are currently under investigation in cancer (118, 119). These are attractive because of their ability to target not only a single gene, but entire pathways (120). Similar strategies capable of inducing these pathways may therefore be efficacious in alleviating autoimmune inflammation. Indeed, MEG3, a long non-coding RNA can modulate TIGIT expression on CD4^+^ T cells from aplastic anaemia patients (121).

The co-expression of checkpoint molecules on individual cells suggests the approach of targeting multiple checkpoints simultaneously, and there has been significant recent interest in targeting dual targets by bi-specific antibodies. CTLA-4/OX40 (ATOR-1015) (122), and PD-L1/CTLA-4 (KN046) bi-specifics are currently in trial for use in cancer (123). In RA, bi-specific approaches have the potential advantage of targeting checkpoint molecules specifically in the inflammatory niche. For instance, a bi-specific targeting TNF and synovial-specific domain scFv-A7, shows remarkable tissue and disease specificity for the microvascular compartment of the human arthritic ST (124). Interestingly, fewer irAEs appear to be observed following the use of bi-specific antibodies in cancer therapy compared to combination therapy, likely as a result of their site-specific nature (125).

Finally, while the adverse events associated with the use of ICIs in cancer therapy are reasonably well documented, the current lack of agonistic antibodies targeted to checkpoint pathways in human trials makes it difficult to remark on the potential risks associated with the use of checkpoint agonists for the treatment of chronic diseases such as RA. In initial trials, severe adverse events were documented while testing an anti-CD28 agonist in healthy control patients (126), while early trials of Peresolimab in RA do not suggest the development of severe adverse events (61), and in cancer, agonistic antibodies targeting OX40 showed acceptable safety profiles (127). However longer, and larger trials are required to fully assess safety profiles and effect on oncologic risk (61).

## 8 Concluding remarks

The role of immune checkpoint molecules in RA is a growing area of research, and the elucidation of how these pathways influence the breaching of self-tolerance and drive RA pathogenesis will undoubtedly be critical for selecting the most appropriate therapy for individual patients. However, much remains to be explored, from deciphering the complex interactions between immune cells to fine-tuning the delicate equilibrium of immune activation and suppression. As this field progresses, agonistic immune checkpoint-based therapies may emerge as valuable therapeutic tools for treating RA and may offer new avenues for improved patient care and quality of life.

## Author contributions

AS: Conceptualization, Writing – original draft, Writing – review & editing. KL: Writing – original draft, Writing – review & editing. MW: Conceptualization, Funding acquisition, Project administration, Resources, Supervision, Writing – review & editing.
